# Complete Blood Count Values Over Time in Young Children During the Dengue Virus Epidemic in the Dominican Republic From 2018 to 2020

**DOI:** 10.1155/2024/3716786

**Published:** 2024-08-03

**Authors:** Melissa E. Day, Yonairy Collado Puello, Miguel E. Mejía Sang, Elvira J. Diaz Brockmans, María F. Díaz Soto, Stephanie M. Rivera Defilló, Karla M. Taveras Cruz, Javier O. Santiago Pérez, Rafael Meña, Cesár Mota, Margaret K. Hostetter, Louis J. Muglia, Javier Gonzalez del Rey, Elizabeth P. Schlaudecker, Lisa J. Martin, Brittany N. Simpson, Carlos E. Prada

**Affiliations:** ^1^ Division of Infectious Diseases Cincinnati Children's Hospital Medical Center, Cincinnati, Ohio, USA; ^2^ School of Medicine Instituto Tecnológico de Santo Domingo, Santo Domingo, Dominican Republic; ^3^ School of Medicine Universidad Iberoamericana (UNIBE), Santo Domingo, Dominican Republic; ^4^ Department of Pediatrics Centro de Obstetricia y Ginecología, Santo Domingo, Dominican Republic; ^5^ Pediatric Emergency Medicine Department Hospital Infantil Dr. Robert Reid, Santo Domingo, Dominican Republic; ^6^ Department of Pediatrics University of Cincinnati College of Medicine, Cincinnati, Ohio, USA; ^7^ Division of Human Genetics Cincinnati Children's Hospital Medical Center, Cincinnati, Ohio, USA; ^8^ Division of Pediatric Emergency Medicine Cincinnati Children's Hospital Medical Center, Cincinnati, Ohio, USA; ^9^ St. Jude Children's Research Hospital Department of Pediatrics University of Tennessee Health Science Center Le Bonheur Children's Hospital, Memphis, Tennesse, USA; ^10^ Division of Genetics Birth Defects & Metabolism Ann & Robert H. Lurie Children's Hospital of Chicago, Chicago, Illinois, USA; ^11^ Department of Pediatrics Feinberg School of Medicine of Northwestern University, Chicago, Illinois, USA

**Keywords:** children, dengue fever, laboratory values, severe dengue

## Abstract

**Background:** Dengue fever (DF) is a mosquito-borne illness with substantial economic and societal impact. Understanding laboratory trends of hospitalized Dominican Republic (DR) pediatric patients could help develop screening procedures in low-resourced settings. We sought to describe laboratory findings over time in DR children with DF and DF severity from 2018 to 2020.

**Methods:** Clinical information was obtained prospectively from recruited children with DF. Complete blood count (CBC) laboratory measures were assessed across Days 1–10 of fever. Participants were classified as DF-negative and DF-positive and grouped by severity. We assessed associations of DF severity with demographics, clinical characteristics, and peripheral blood studies. Using linear mixed-models, we assessed if hematologic values/trajectories differed by DF status/severity.

**Results:** A total of 597 of 1101 with a DF clinical diagnosis were serologically evaluated, and 574 (471 DF-positive) met inclusion criteria. In DF, platelet count and hemoglobin were higher on earlier days of fever (*p* < = 0.0017). Eighty had severe DF. Severe DF risk was associated with thrombocytopenia, intraillness anemia, and leukocytosis, differing by fever day (*p* < = 0.001).

**Conclusions:** In a pediatric hospitalized DR cohort, we found marked anemia in late stages of severe DF, unlike the typically seen hemoconcentration. These findings, paired with clinical symptom changes over time, may help guide risk-stratified screenings for resource-limited settings.

## 1. Introduction

Dengue virus infection is the most prevalent mosquito-borne illness affecting human populations [1, 2]. Over 3.6 billion individuals live in areas amenable to dengue virus infection [2]. The 2019–2020 dengue outbreak resulted in the largest number of dengue cases ever reported globally [3]. In 2019, the Region of the Americas reported the largest annual incidence of dengue fever (DF) at 3.1 million with 28,000 cases of severe DF [3]. The disease's economic and social impact has led to a steady rise in disability-adjusted life-years from 1990 to 2013, especially in the Region of the Americas with an almost 300% increase by 2013, the largest increase seen across studied regions [2].

DF can lead to significant laboratory value derangements, particularly later in the disease course, including leukopenia (white blood cell [WBC] < 5000 cells/mm^3^), thrombocytopenia (100–150,000 cells/mm^3^), and mild increases in hematocrit (by 10%), with more severe thrombocytopenia and rising hematocrit in severe DF [4]. However, initial values may be normal [4], making it difficult for low-resourced facilities to differentiate DF from other illnesses due to expense and time involved with serologic testing. While trends in blood count values in DF patients have been described in Asian countries such as Bangladesh [5] and Thailand [6], baseline rates of anemia in these countries are different compared to the Dominican Republic (DR). In 2019, Bangladesh had a reported prevalence of anemia in children less than 5 years of 43%, compared to only 28% of children in the DR, which may impact blood count values associated with DF at presentation [7]. Furthermore, a prior study in the DR revealed that anemia was significantly associated with severe DF in a pediatric cohort [8], unlike the more common hemoconcentration within this population [4]. Additionally, the DR is geographically distinct from other, more well-studied areas, and understanding the associated blood count trends in this disease within the context of this unique country could help develop future screening procedures in low-resourced settings. Furthermore, given the dramatic rise in cases in the Americas in recent years, it is imperative to further the knowledge of this region's DF presentation and trends, especially with increasing transmission and expanded areas amenable to DF infection given climate change [9].

To address the need for enhanced DF identification and risk stratification [5] within the Regions of the Americas, we aimed to prospectively characterize an observational pediatric cohort in the DR by describing clinical laboratory findings and clinical risk factors in children for DF and DF severity.

## 2. Methods

### 2.1. Study Design

Following study approval (#2018-3656), formal written consent or assent was obtained from guardians or participants (as appropriate). An observational cohort study enrolled children presenting to the Robert Reid Children's Hospital (RRCH) in Santo Domingo, DR, from August 2018 to March 2020 [10]. The study was suspended due to the COVID-19 pandemic. Children were hospitalized for various durations, depending on clinical severity and individualized need for hospitalization. Per the World Health Organization (WHO) 2009 Dengue Guidelines, clinical suspicion for DF includes acute fever for 2–7 days and 2+ of the following symptoms: nausea, vomiting, rash, myalgias, arthralgias, headache, retro-orbital pain, petechiae, positive tourniquet test, or leukopenia [11]. Upon presentation from referring centers, patients were triaged in the emergency department, then admitted to the RRCH-dedicated DF ward, step-down unit, or pediatric intensive care unit (PICU) [8]. Patients had plasma anti-DF immunoglobulins (IgM) assessed through public government surveillance mechanisms via ELISA to determine acute dengue viral infection, as previously described [10]. ELISA testing was performed on the day of admission, if reagents were available for testing as part of routine patient care. Individuals with clinical signs suggestive of DF but negative testing were considered DF-negative during their hospitalization. DF viral testing returned after hospitalization due to the national surveillance system's laboratory protocols. Samples were obtained with admission but were not available for clinical management as they could return after a patient's discharge. Participants received care based on clinical opinion.

### 2.2. Data Collection and Analyses

Demographics, clinical history, and objective data were obtained via chart review. All laboratory data represented routine management at RRCH, staffed by DF experts [8]. Per their typical protocol, labs, including complete blood counts (CBCs), were acquired at admission, and daily thereafter. However, depending on the clinical picture and need, laboratory studies could be repeated throughout the day. Studies obtained outside of RRCH by outside and/or referring hospital laboratory values were included also in the study, if available.

### 2.3. Hematologic Studies

CBC values were assessed over day of fever (DoF) 1–10, as available: hemoglobin (g/dL), hematocrit (%), mean corpuscular volume (MCV, fL), mean corpuscular hemoglobin concentration (MCHC, g/dL), red blood cell distribution width (RDW, %), WBC count (×10^3^/mm^3^), platelet count (×10^3^/mm^3^), monocyte count/% (×10^3^/mm^3^; %), neutrophil count/% (×10^3^/mm^3^; %), and lymphocyte count/% (×10^3^/mm^3^; %).

### 2.4. Study Definitions

Participants were classified based on the WHO 2009 criteria into children without warning signs, children with 1+ warning signs, and severe DF [6]. Warning signs include abdominal pain/tenderness, persistent vomiting, clinical fluid accumulation, mucosal bleeding, lethargy/restlessness, liver enlargement > 2 cm, and increased hematocrit with a rapid decrease in platelet count. Those with severe DF were defined by presence of shock, fluid accumulation with respiratory distress, critical bleeding, and end-organ damage [11]. Gastrointestinal (GI) symptoms included abdominal pain, vomiting, diarrhea, or hepatomegaly. Rash included petechial findings.

DoF was defined as the onset of fever per the history upon presentation. If a child or family member reported that no fever had occurred upon presentation, then the DoF first seen in the hospital was recorded as the first DoF.

### 2.5. Statistical Analyses

Due to distribution, RDW, WBC, platelet count, monocyte count and percentage, neutrophil count and percentage, and lymphocyte count underwent natural log transformation. Data were summarized using means and standard deviations for continuous variables, and frequencies and percentages for categorical variables. Participants were divided by IgM titer results as DF-negative and positive. For analysis purposes and stratification of factors related to the highest risk group, severe dengue, participants were classified into two groups: nonsevere DF (encompassing children without and with 1+ warning sign(s)) and severe DF. We assessed the association of DF severity with demographics, clinical characteristics, and peripheral blood studies utilizing chi-squared goodness of fit or Kruskal–Wallis (Rank Sums) tests. Using linear mixed-models, we assessed if hematologic values differed by DF status and over time, including age and sex as covariates. For type-specific WBC, the total WBC count was included as a covariate. To account for multiple testing, *p* ≤ 0.0033 was considered significant.

## 3. Results

### 3.1. Baseline Characteristics

**Figure 1:**
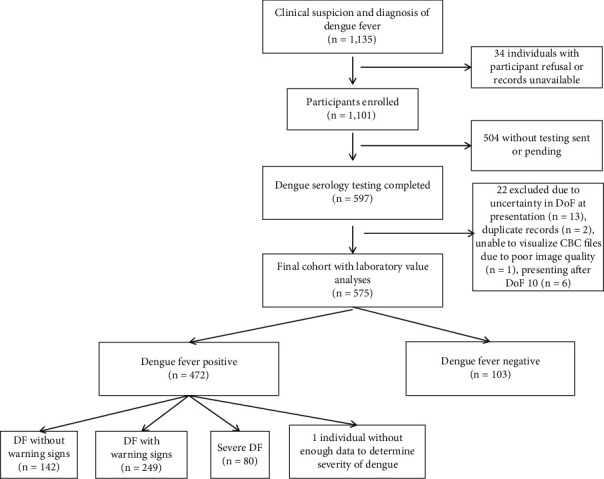
Study flow diagram for participant enrollment.

From 2018 to 2020, 1135 individuals were approached for our study with 34 declining or having no record available. Of the participants (*n* = 1101), 597 had DF serology testing completed. Individuals with testing completed were more likely to occur earlier in the study time frame. Focusing on laboratory study analyses, we then excluded 13 individuals due to uncertainty in DoF at presentation, two due to duplicate records, one due to inability to visualize CBC files from poor image quality, and one due to lack of enough information to classify severity of dengue. Given the focus of analysis on DoF 1–10, six additional participants were excluded as they presented after DoF 10. Final sample included 574 participants including 103 without DF and 471 with DF. Using the 2009 WHO Dengue Guidelines [11], 142/471 (30%) had no warning signs, 249/471 (53%) had warning signs, 80/471 (17%) had severe DF.  Figure 1 details a flowchart of participants who were included and excluded.

Within the full DF-positive cohort with severity data (*n* = 471), our participants have a median age of 59 months (range 13–88 months) with 49% female. Figure S1 displays the number of cases per week in 2019 with the severe DF group highlighted. As previously reported [10], four participants succumbed due to DF complications with a case-fatality rate of 0.082%, overall similar to the reported 0.049% within the Region of the Americas during 2019 [3]. These four participants had overall lower hemoglobin and higher WBC counts compared to others in the PICU. Individuals with severe DF had no difference between or across sex, age, GI symptoms, presence of rash, or presence of headache compared to the nonsevere group (Table 1).

### 3.2. CBC Findings Across DoF

#### 3.2.1. DF Versus No DF

**Figure 2:**
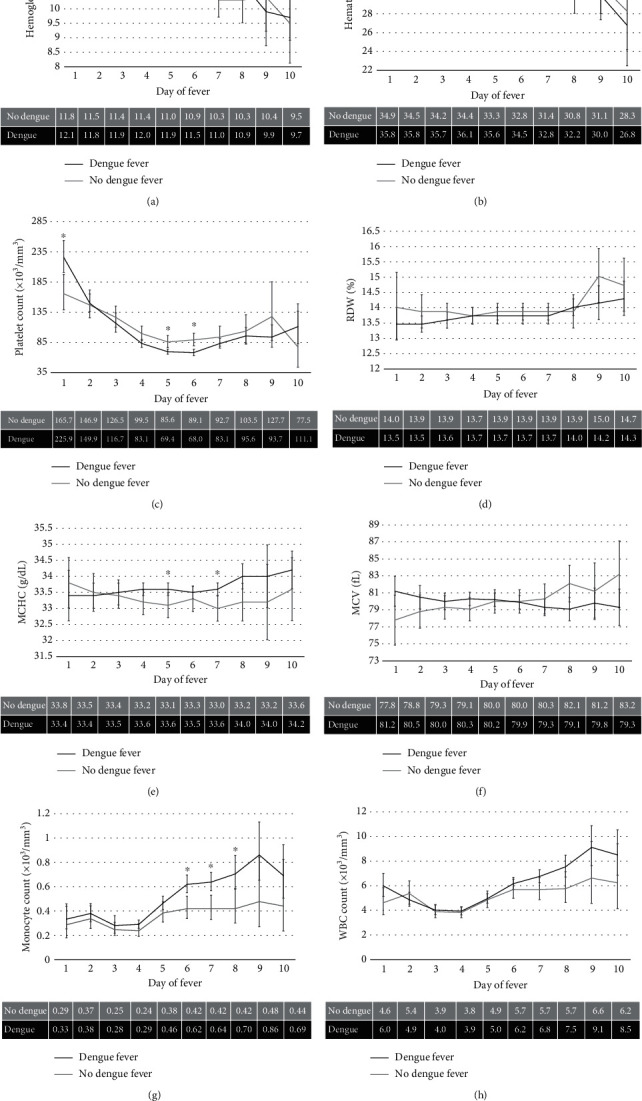
CBC values across day of fever in DF (black) and non-DF (gray) patients: (a) hemoglobin, (b) hematocrit, (c) platelet count, (d) red blood cell distribution width (RDW), (e) mean corpuscular hemoglobin concentration (MCHC), (f) mean corpuscular volume (MCV), (g) monocyte count, and (h) white blood cell (WBC) count. The mean values for each value across day of fever are listed below each graph. Values with an asterisk (∗) represent significant values (*p* ≤ 0.0033).

Platelet count was significantly higher on DoF 1 (*p* = 0.0017) and lower on DoF 5 and 6 (*p* = 0.001 and *p* < 0.0001, respectively) in those with DF compared to those without DF. Individuals with DF had platelet levels which dropped more quickly across DoF for hematocrit and hemoglobin (*p* ≤ 0.002). Those with DF had higher hemoglobin levels earlier in the disease course, especially DoF 4–5 (p  = 0.0009 and *p* < 0.0001, respectively). Those with DF had increasing levels of MCHC, monocyte number, and WBC counts across DoF (*p* ≤ 0.003). Additionally, DF-positive individuals showed increasing RDW and MCV while DF-negative individuals showed decreasing values (*p* ≤ 0.0003, *p* ≤ 0.0001, respectively). WBC count, RDW, and MCV did not vary across DoF in those with or without DF (Figure 2).

#### 3.2.2. Severe DF Versus Nonsevere DF

**Figure 3:**
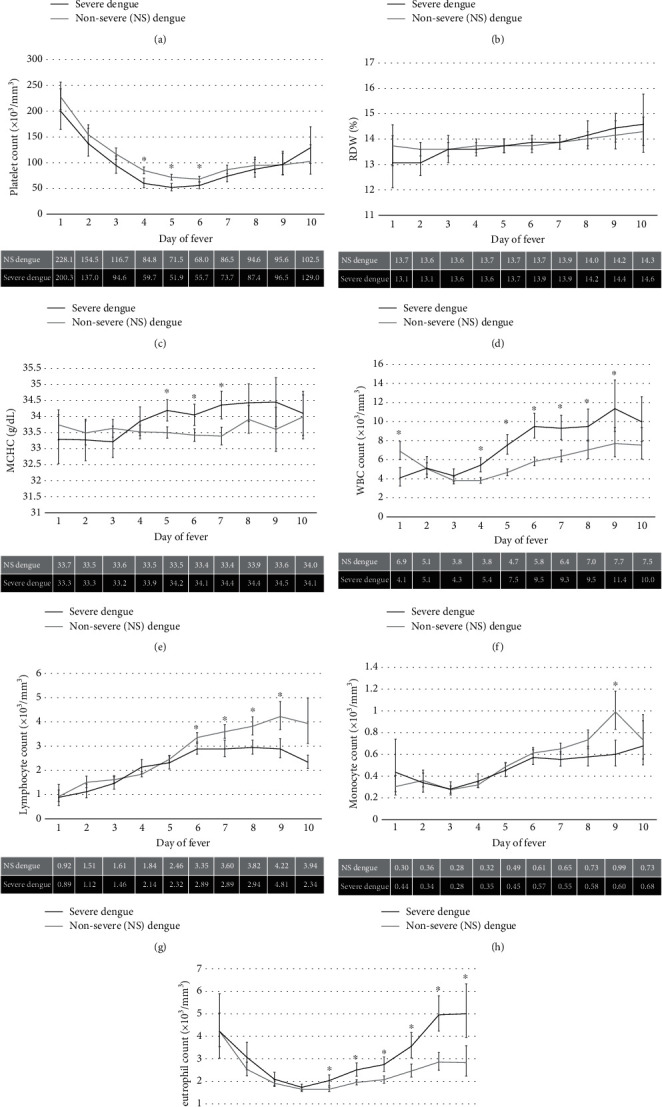
CBC values across day of fever in severe DF (black) and nonsevere (NS) DF (gray) patients: (a) hemoglobin, (b) hematocrit, (c) platelet count, (d) red blood cell distribution width (RDW), (e) mean corpuscular hemoglobin concentration (MCHC), (f) white blood cell (WBC) count, (g) lymphocyte count, (h) monocyte count, and (i) neutrophil count. The mean values for each value across day of fever are listed below each graph. Values with an asterisk (∗) represent significant values (*p* ≤ 0.0033).

Compared to nonsevere DF, children with severe DF had significantly lower hemoglobin and hematocrit on DoF 7–9 (*p* ≤ 0.0005) with levels dropping more quickly across DoF for both measures (*p* < 0.0001). Likewise, for severe DF, MCHC was significantly higher on DoF 5–7 (*p* ≤ 0.0004) with levels increasing more quickly for MCHC. An increase in RDW across time was also seen in severe DF (*p* = 0.0004). Platelet counts were lower on DoF 4–6 in severe DF (*p* < 0.0001). WBC counts were significantly lower on DoF 1 (*p* = 0.0002) and higher on DoF 4–9 (*p* ≤ 0.0025), lymphocyte counts were significantly lower on DoF 6–8 (*p* ≤ 0.0010), neutrophil counts were significantly higher on DoF 5–10 (*p* ≤ 0.0006), and monocyte counts were lower on DoF 9 (*p* < 0.001, Figure 3).

## 4. Discussion

DF is an increasing global health concern, with the 2019 dengue epidemic highlighting its impact. In this study of a young pediatric hospitalized population in the DR, CBC values were associated with DF status and severity in Dominican children. Specifically, DF+ individuals had higher hemoglobin levels on earlier DoF with decreases later in disease along with increasing RBC indices over time. Furthermore, decreasing RBC measures and differential changes in WBC subpopulations characterized severe DF, inconsistent with typical RBC hemoconcentration traditionally reported. Leukocytosis with neutrophilia also characterized the later disease course within the severe DF group. Platelet measures followed the typical downward trajectory over time in both DF+ and severe DF participants, though platelets were elevated at presentation in those with DF. Providers may consider these factors and associated clinical symptoms for risk stratification and identification of DF when caring for children in endemic areas presenting with DF signs but limited ability to obtain confirmatory testing.

Assessment of CBC values across DoF revealed significant differences based on DF status in DR children. Compared to non-DF individuals, children with DF had higher hemoglobin and hematocrit values around DoF 4–5 and significant thrombocytopenia on DoF 5–6, consistent with the WHO definition [4]. However, we report a higher platelet count in individuals with DF compared to no DF on DoF 1, in contrast to prior reports of normal platelet counts or mild thrombocytopenia [4]. A possible explanation may be that platelet count rises as an acute-phase reactant during the acute febrile phase of DF.

In regard to RBC measures, those with severe DF experienced a sustained decrease in RBC mass across DoF, opposite to expected [10–13] but consistent with previous investigations at this hospital [8]. Those who succumbed to illness had lower hemoglobin and hematocrit counts than others in the PICU. Hemoglobin and hematocrit began to decrease around Day 5, similar to previous reports, but they remained low, a stark contrast to the sudden rise typically noted following a drop in platelet counts, thought to be due to plasma leakage [11]. Additionally, anemia has been associated with severe DF in Brazilian children [11, 14]. From a prior study from this hospital, we know that the observed anemia is not due to concomitant malaria [15]. Anemia could suggest chronic suboptimal nutrition, a risk factor for severe DF, as we know that over one in four children less than 5 years old in the DR have anemia [7]. Despite a more prevalent incidence of anemia in Asian countries [7], individuals within our cohort appear to be more susceptible to severe dengue and show lower RBC mass later in the illness despite less anemia overall. Anemia could also be due to early volume replacement and bleeding, thus requiring further studies into interventional factors [14].

The physiological explanations for the trends in blood count values over time shed light on the importance of laboratory monitoring for dengue-related morbidity and mortality. The sustained anemia that participants with severe DF had is likely due to progression of plasma leakage with fluid loss into third spaces, as severe DF can involve hemorrhagic fever and shock [11]. Routinely assessing hemoglobin levels during admission would allow for risk assessment for severe plasma leakage and subsequent mortality. The neutrophilia encountered later in the severe DF disease course likely reflects either a superimposed bacterial process or ongoing systemic inflammation [16], which could be used as a marker to assess for severity of disease and possible comorbidities. Overall, our laboratory value findings highlight the role of active surveillance of blood count values, particularly hemoglobin, over time in order to promptly administer therapies such as blood transfusions, fluid management, or antibiotics in order to reduce mortality.

Development of a cheap, rapid, and specific test is greatly needed and should be prioritized given increasing DF incidence [1] and current challenges of identifying those at highest risk [17]. Our results add to the general knowledge of laboratory value abnormalities over disease course in young children suspected to have DF, highlighting a country in the Region of the Americas with rising rates of DF. Given associations between climate change and dengue transmission due to increased precipitation, higher temperatures, and times of drought [18], further understanding of factors surrounding DF diagnoses in this region is necessary. Further studies are needed to develop improved, more easily accessible diagnostics in resource-limited settings, where serology or other specific testing is limited or unavailable. Resource limitations are further stretched given the nontrivial cost of care for individuals with DF with warning signs, a group at risk for progression and needing monitoring, encompassing 50% of those with symptomatic DF [11, 19].

Our study has some limitations. Our population only captures children requiring admission and monitoring, limiting generalizability. However, risk stratification within this population is most needed [20]. The lack of DF diagnostic testing on a high percentage of individuals (46%, *n* = 504) highlights the continued healthcare strain and the access barriers within DF-endemic countries, despite WHO recommendation for all cases to have laboratory confirmation [11]. The limited availability of serology testing was likely influenced by the rapid increase in cases during the 2018–2020 epidemic. Given similar constraints, it was unclear if participants were experiencing an initial infection or subsequent reinfection from DF. Additionally, because our study was only able to determine the presence or absence of clinical symptoms before or during hospitalization to appropriately categorize dengue diagnoses, we were unable to evaluate the evolution of symptoms and their associated clinical management over the course of the hospitalization and therefore unable to pair these observations with CBC values, which will be a key next step for future studies.

## 5. Conclusion

In conclusion, DF is an increasing, global problem with a growing footprint on millions of lives. At this time, monitoring decreases in hemoglobin and increases in WBC counts, particularly neutrophils, through routine CBC testing in hospitalized patients with suspected DF may identify those young children at higher risk of severe DF and death in the DR. Our study's significant percentage of excluded individuals without diagnostic testing highlights the need for screening algorithms utilizing laboratory and clinical symptoms to diagnose and guide care of patients with DF in low-resourced settings, where other forms of testing are either expensive, resource-intensive, or have long turnaround times. Given the increasing burden of infectious diseases worldwide, further research is needed to help curb the impacts of dengue viral infections on health and society. Future studies are needed to examine the changes in clinical symptoms and management over time in relation to changes in blood count values and how these may change clinical classifications of DF diagnoses.

## Figures and Tables

**Table 1:** Population characteristics by dengue fever severity.

	**Total DF-positive (** **n** = 471**)**	**Nonsevere DF (** **n** = 391**)**		**Severe DF (** **n** = 80**)**	***p* value**
		**Without warning signs (** **n** = 142**)**	**With warning signs (** **n** = 249**)**		
Age (months (median IQR))	59 (13-88)	54 (11.8–88)	62 (19–94)	59 (10–77.5)	0.37
Age group (*N* (%))					0.35
Less than 1 year	109 (23%)	35 (25%)	51 (21%)	23 (29%)	
1 to less than 5 years	128 (27%)	40 (28%)	70 (28%)	18 (23%)	
5 years and greater	233 (50%)	67 (47%)	127 (51%)	39 (49%)	
Female	229 (49%)	67 (47%)	119 (48%)	43 (54%)	0.31
Presence of GI symptoms	349 (74%)	90 (63%)	198 (80%)	61 (76%)	0.65
Age group (*N* (%))					
Age less than 1 year		18 (51%)	30 (59%)	17 (74%)	0.11
1 to less than 5 years		23 (58%)	57 (81%)	12 (67%)	0.60
5 years and greater		49 (73%)	111 (87%)	32 (82%)	0.95
Rash	99 (21%)	28 (20%)	57 (23%)	14 (18%)	0.38
Age group (*N* (%))					
Age less than 1 year		12 (34%)	23 (45%)	8 (35%)	0.60
1 to less than 5 years		8 (20%)	14 (20%)	1 (6%)	0.19
5 years and greater		8 (12%)	20 (16%)	5 (13%)	0.79
Headache	87 (19%)	21 (15%)	53 (21%)	13 (16%)	0.56
Age group (*N* (%))					
Age less than 1 year		0 (0%)	2 (4%)	0 (0%)	1.0
1 to less than 5 years		1 (2.5%)	9 (13%)	3 (17%)	0.39
5 years and greater		20 (30%)	42 (33%)	10 (26%)	0.43

## Data Availability

The clinical data used to support the findings of this study have not been made available, in order to maintain patient privacy and confidentiality.

## Supporting Information

Supporting Information Additional supporting information can be found online in the Supporting Information section. Figure S1 Total number of DF-positive participants shown on the *x*-axis per epidemiologic week (*y*-axis). A number of severe cases are highlighted separately by the black bars per epidemiologic week of 2019.
