# Supplementary material for: Complete Blood Count Values Over Time in Young Children During the Dengue Virus Epidemic in the Dominican Republic From 2018 to 2020
Source: Biomed Res Int. 2024 Aug 3;2024:3716786. doi: 10.1155/2024/3716786 (PMC11316910; doi:10.1155/2024/3716786)
Supplement: Supporting Information — Additional supporting information can be found online in the Supporting Information section. Figure S1 Total number of DF-positive participants shown on the x-axis per epidemiologic week (y-axis). A number of severe cases are highlighted separately by the black bars per epidemiologic week of 2019. [file 3716786.f1.docx]

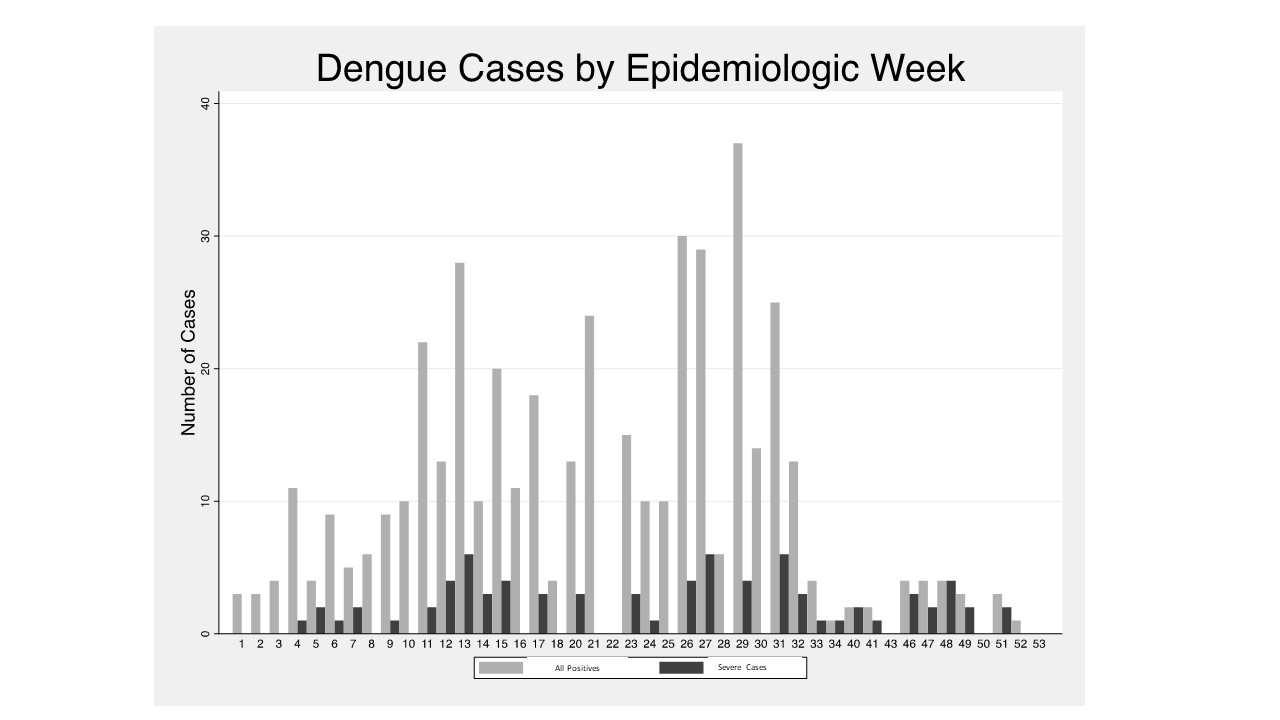


**Supplemental Figure 1.** Total number of DF positive participants shown on the X-axis per epidemiologic week (Y-axis). Number of severe cases are highlighted separately by the black bars per epidemiologic week of 2019.
